# A Protocol to examine the needs and experiences of autistic children/young people or children/young people with an intellectual disability when receiving support for mental health difficulties from child and adolescent mental health services

**DOI:** 10.12688/wellcomeopenres.23597.2

**Published:** 2025-06-25

**Authors:** Naomi Williams, Julie Taylor, Helena Tuomainen

**Affiliations:** 1 Health Sciences, Warwick Medical School, University of Warwick, Warwick, England, CV4 7AL, UK; 2 Nursing and Midwifery, School of Health Sciences, University of Birmingham, Birmingham, England, B15 2TT, UK

**Keywords:** Autism; Intellectual Disability; CAMHS; Child and Adolescent Mental Health Services; Children and Young People; Mental Health Difficulty; Narrative Interviews; Parents; Carers; Youth Voice; Needs; Experiences; Storytelling; Referrals; Assessment; Psychological Therapy; Neurodevelopmental Difference; Neuro-difference

## Abstract

**Background:**

Studies indicate a high prevalence of co-occurring mental health conditions in autistic children and young people (CYP) and CYP with intellectual disability (ID), with approximately 25% experiencing mental health conditions compared to 17.2% of their non-autistic or non-ID counterparts. Delivering psychological therapies to autistic CYP and CYP with ID proves challenging due to inaccessible mental health provision. Further research is necessary to comprehend autistic CYP and CYP with ID and their parent/carers needs and experiences within mental health services and identify barriers to access and engagement. The perspectives of autistic CYP or CYP with ID and their parent/carers should be incorporated into treatment planning to enhance psychological therapies.

**Methods:**

We will conduct between 1–3 Narrative Interviews per participant, lasting up to 1.5 hours per ‘sitting’. Narrative interviews are a qualitative research method used to collect detailed and thorough information from individuals about their life events, insights, and perspectives. The study will involve CYP creating metaphors, allowing them to share their story. The study will use alternative methods of communication specific to their needs to allow the research team to explore the complexities of autistic CYP and CYP with ID needs and experiences. Additionally, parents/carers of autistic CYP and CYP with ID will tell their story of the needs and experiences of their child verbally.

**Results:**

This study results will help to share the needs and experiences of the autistic CYP and CYP with ID, or a co-occurrence of both diagnoses and expose the barriers to accessing mental health services for autistic CYP and CYP with ID.

**Conclusions:**

Recommendations will include guidance on decision-making for referrals, assessment, and treatment provision for autistic CYP and CYP with ID and co-occurring mental health difficulties.

## Plain Language Summary

Children and young people with a neurodevelopmental difference, such as autism or intellectual disability, are 4.5 times more likely to experience anxiety or depression than their peers, known as a co-occurring mental health difficulty. Furthermore, 27.4% of referrals to mental health services in 2023–24 were children and young people under 18, with a neurodevelopmental difference, emphasising the need for early intervention from mental health services. Autistic children and young people and children and young people with intellectual disability are either told that they do not meet the criteria for mental health provision or find it difficult to engage with the psychological therapies offered following the assessment stage. This study will carry out narrative interviews with autistic children and young people, children and young people with intellectual disability and their parents or carers, where individuals can share their stories, including needs and experiences when using mental health services. The study supports the government's emphasis on early intervention and prevention of mental health difficulties. It will provide valuable recommendations for future service planning and mental health provision strategies for autistic children and young people and children and young people with intellectual disability.

## Introduction

In England, approximately 1–2% of children are autistic ( Underwood *et al.*, 2022), and around 2.5% of children have ID ( Patel *et al.*, 2020). Both conditions fall within the category of neurodevelopmental conditions and typically manifest during childhood ( Howlin, 2000). According to the DSM-5 diagnostic criteria, autism is characterised by persistent deficits in social communication and interaction, as well as restricted or repetitive behaviours ( American Psychiatric Association, 2013; Carrington *et al.*, 2014). ID is defined by the ICD-11 as a group of developmental conditions characterised by a significant impairment of cognitive functions, which are associated with limitations of learning, adaptive behaviour and skills ( Bertelli *et al.*, 2016). Both autism and ID can be classified into different levels of severity, ranging from mild to severe. However, this study focuses on mild and moderate presentations of autism or ID, those who have either one or both conditions. The information may not apply to individuals with severe autism or ID, and further research is needed to address their specific needs.

Additionally, everyone with a diagnosis of autism or ID, or co-occurrence of both, is unique, and the descriptions provided may not fully align with the experiences or preferred language of every child and young person in this population. Mental health plays a vital role in overall well-being and mental health difficulties can disrupt cognitive, emotional, or behavioural processes.

## Rationale

It is common for autistic CYP and CYP with ID to be refused a mental health service based on perceived complexities following a referral to CAMHS ( Babalola *et al.*, 2024). This results in autistic CYP or CYP with ID being signposted to specialist disability services, such as autism or intellectual disability services, rather than services specifically for their mental health difficulty due to diagnostic overshadowing ( Ashworth *et al.*, 2025). This leads to no or limited interventions for mental health difficulties, such as low mood, anxiety, OCD and panic disorder. This can often create more complex mental health problems where autistic CYP and CYP with ID feel that they are “broken” and “cannot be fixed” as shared by patient and public involvement (PPI) parent/carer groups during consultations on this study in 2023. A review found that autistic children in the UK experience system-level barriers, stigma and patient-provider-parent communication issues ( Babalola *et al.*, 2024) similar to the findings of a review for those with ID ( Whittle *et al.*, 2017). As a result of not intervening, early referrals to child and adolescent mental health services (CAMHS) crisis teams or accident and emergency attendances approximately 6–12 months later are likely. It is probable that if clinicians intervened early, then the individual’s mental health may not have deteriorated. A qualitative study capturing the lived experiences and perceived needs of this population and their families can reveal specific barriers, such as communication difficulties, service inflexibility, or sensory environments that quantitative data alone can often overlook. By identifying these nuanced, contextual factors, the study can inform the design of more accessible, responsive, and equitable services. In turn, this can support earlier intervention, reduce disengagement, and ultimately contribute to improved mental health outcomes for this underserved group. The study will better understand the needs and experiences of autistic CYP or CYP with ID, and the views of parents or carers. This study will help evidence the voice of the autistic CYP and CYP with ID and expose the barriers to access for autistic CYP and CYP with ID. Recommendations will include guidance on decision-making on referrals, assessment, and treatment for autistic CYP and CYP with ID and co-occurring mental health difficulties. Incorporating lived experiences into service planning aligns with person-centred care, national policies and priorities around co-production, and inclusivity in health care delivery.

The rationale for including both autistic CYP and CYP with intellectual disability was based on:

1. The significant clinical overlap and co-occurrence of these conditions, approximately one-third, 37.9% of autistic children also have intellectual disability (Centers for Disease Control and Prevention, 2023).
2. The shared challenges both groups face in accessing appropriate mental health support.
3. Similar adaptations are often needed to make psychological therapies accessible to both populations.

## Research aims/questions/objectives

### Aims

1) To understand the needs of autistic CYP and/or CYP with ID when accessing support for mental health difficulties.
2) To understand the experiences of autistic CYP and/or CYP with ID when receiving support for mental health difficulties from mental health services.

### Questions

1) What are the needs of autistic CYP and/or CYP with ID and their parents/carers when accessing mental health support?
2) What are the experiences of autistic CYP and/or CYP with ID and their parents/carers when accessing mental health support?

### Objectives

1) To examine the time intervals between key stages in mental health care pathway, from the onset of a mental health difficulty, referral, triage, assessment, intervention and discharge for autistic CYP and/or CYP with ID.
2) To identify and explore barriers faced by autistic CYP and those with ID face in accessing timely and appropriate mental health services.
3) To evaluate the perceived and actual impact of mental health services on outcomes autistic CYP and those who have ID.

## Methods

### Narrative interview development

The research team, alongside PPI members and stakeholders, conducted iterative consultations to develop the topic guide and research materials for the narrative interviews.

### Study design and methods of data collection and analysis

There will be between 1–3 qualitative narrative Interviews per participant, lasting up to 1.5 hours per ‘sitting’. Throughout this paper, the term ‘sitting’ will refer to one interview and will account for reasonable adjustments for pauses, breaks, or rescheduling if needed. Narrative interviews are a qualitative research method used to collect detailed and thorough information from individuals about their life events, insights, and perspectives. Unlike closed, semi-structured, and open-ended interviews, narrative interviews will encourage autistic CYP and CYP with an ID, their parents/carers to share their stories openly in a conversational manner. Previous studies ( Groleau *et al.*, 2006) have used narrative interviews with parents/carers, autistic CYP and CYP with an ID, and they have proven to be effective in eliciting information and free flow of conversation. The study will use alternative methods of communication specific to their needs to allow the research team to explore the complexities of autistic CYP and CYP with ID needs and experiences using creative methods that will help them engage with the interview topic guide. Alternative methods of communication include Makaton, symbols, pictures, photos, and social stories to ensure accessibility and inclusion. The study will involve CYP creating metaphors ( Mattingly & Lawlor, 2000), allowing them to share their story, such as creative methods including art, drawing, dolls, teddies, sensory, sand with dinosaurs or figures, legos, imaginative play, poems, mood boards/collages using magazine cuttings, rocks/flowers and talking mats to symbolise and communicate experiences. Additionally, parents/carers of autistic CYP and CYP with an ID will be interviewed and encouraged tell their story of the needs and experiences of their child verbally. Narrative interviews with autistic CYP and CYP with ID will be conducted to the point of trustworthiness and reliability ( Hennink & Kaiser, 2022). Although the study includes a small sample size, the method used will collect detailed and thorough information from individuals about their life events, insights, and perspectives ( Bekele & Ago, 2022). Unlike closed, semi-structured, and open-ended interviews, narrative interviews will encourage autistic CYP and CYP with an ID, and/or their parents/carers to share their stories openly in a conversational manner.

### Interview topic guide

***Topics***

•   Signposting advice for support services following diagnosis of disability or additional need

•   Mental Health Difficulty

•   CAMHS referral and or assessment process

•   CAMHS treatment process using a psychological therapy

•   Communication with child or parent

•   Development of rapport with child or parent

•   How knowledgeable and confident clinicians are

### Narrative interview parent/carer (prompts)

Consideration has been taken on whether both parents or one parent will be invited to the interviews. I will take a flexible approach to allow the parents or carers to exercise autonomy. However, it will be noted that the difference in viewpoints and events can affect the direction of the experience being shared. Further development will be through input from the narrative interviews before data collection, as such interviews will be conducted over time.

Outline the purpose, structure of each interview and key themes.

- Start with -

Tell me about the child (a typical day in the life of…)

Signposting advice for support services following diagnosis of disability or additional need

When noticed a mental health difficulty

Mental Health Difficulty

- Next

CAMHS referral and or assessment process (patient journey)

CAMHS treatment process using a psychological therapy (patient journey)

- Finally

Communication with child or parent

Development of rapport with child or parent

How knowledgeable and confident clinicians are (patient journey)

### Narrative interview child (prompts)

The aim is for the child to be able to share the story and guide the direction through play. The interviewer will only ask questions or prompts based on the story being told. There will be several options for CYP to choose from when engaging in narrative interviews.

Outline the purpose, structure of each interview and key themes using a social story and/or video animation explaining the process. Offer a choice of play: art, drawing, dolls, teddies, sensory, sand with dinosaurs or figures, Lego, imaginative play. Older CYP may choose to use a storyboard with magazine cuttings to represent their journey or write a poem.

- Start with -

Build character in story: Tell me about the character (setting the scene)

Bring in emotions prompts: When noticed a mental health difficulty (patient journey)

- Next, continue the play story, remind the child of the previous story with photos taken or videos and summarise

A trip to the helper: CAMHS referral and or assessment process (patient journey)

Help received: CAMHS treatment process using a psychological therapy

- Finally, summarise the previous story again

Understanding: Communication with child or parent

Relationship: Development of rapport with child or parent

Environment and knowing the child needs: How knowledgeable and confident clinicians are (patient journey)

### Participant recruitment

***Setting***

The study focuses specifically on CAMHS rather than all mental health support services. We recognise the geographical variations in CAMHS setup and delivery, therefore our recruitment strategy deliberately seeks participants from diverse geographical areas across England to capture this variability.

***Eligibility criteria***

Participants will include autistic CYP and/or those with ID aged 5–16, and/or their parents/carers. The age of participants was selected to capture the onset of a mental health difficulty, which typically emerges in early childhood and adolescence and persists into adulthood ( Jones, 2013). This age range will also ensure that the selected measures are developmentally appropriate.

**Inclusion criteria** for CYP: a clinical diagnosis of autism and/or ID; a mental health and/or behavioural difficulty evidenced by the completion of SDQs/DBC to confirm symptoms; age 5–16; either currently or previously receiving psychological therapy treatment through CAMHS; or past experience of unsuccessfully accessing CAMHS even if they may now have accessed CAMHS; reside in England; all ethnicities. Inclusion criteria for parents/carers: Their CYP meets the aforementioned inclusion criteria. This "or" criterion provides flexibility to capture diverse experiences, including both successful and unsuccessful pathways, without requiring participants to have experienced both scenarios.

CYP will be excluded from the study if they are under 5 years of age or over 16 years of age; they do not have a confirmed diagnosis of autism or ID. Parents/carers will be excluded if they are not a parent/carer of an autistic CYP and/or CYP with ID.

***Recruitment target***

The recruitment target is 7–12 autistic CYP or CYP with ID and 7–12 parents/carers of autistic CYP or CYP with ID, a total of 14–24 participants. The selected sample size reflects the rarity and geographic dispersion of ID. Final participant numbers will depend on recruitment success within the constraints of the recruitment timeline and saturation. Recruitment considers those who dropout, a change in circumstances and those who do not wish to be interviewed more than once.

***Recruitment technique***

The present study will primarily utilise purposive sampling to recruit participants. Autistic CYP and/or CYP with an ID and their parent or carer will be identified and screened by a member of the research team and will be recruited through social media, conferences, digital platforms, and existing databases until the target number of participants is reached. Posters and leaflets will be shared on social media platforms (e.g., Twitter, Facebook) held by a member of the research team. The research team will attend family events and conferences to promote the study. Posters and leaflets will be shared on social media platforms (e.g., Twitter, Facebook) held by a member of the research team. The research team will attend family events and conferences to promote the study.

### Procedure summary

Contact will be made with the relevant community, charities, organisations and individuals. The research team will share the invitation letter, study flyer, participant invitation sheet and consent forms with participants via email.

***Communication aids***

In preparation for the narrative interviews, specific communication aids, including a participant information sheet for parents/carers and an animated video ( Williams, 2024)/social story booklet, pictorial consent form, for children, explaining the research and what will happen during the narrative interview using Makaton signs. An individualised communication booklet will be developed in collaboration with each parent/child to offer scaffolding for developing the narrative story alongside the topic guide. Talking Mats and creative play will be used during the narrative interviews as a method for facilitating the narrative interviews for autistic CYP or CYP with ID.

***Locations***

Interviews will be conducted either within the participant’s home, adhering to the risk assessment and policies for conducting research in the home environment, online or within a neutral environment chosen by the participant to ensure ease of access and that the participant is comfortable. Remote interviews will be conducted in a confidential room, through Microsoft teams if this is something that the participant would feel more comfortable with. Consideration has been given to how each environment may influence participant responses. For instance, conducting interviews in the home may enhance comfort and openness, while also reducing the stress associated with travel and competing commitments. In contrast, a neutral venue may help minimise distractions and familial influence but could potentially increase anxiety or sensory overwhelm for autistic CYP or CYP with ID. Online participation, while potentially limiting the depth of responses due to the virtual format, may offer greater convenience, particularly for parents, by allowing engagement at more suitable times, such as when children are asleep or otherwise occupied. All travel for the purposes of the study will be reimbursed. Some participants may opt for completing an interview in one ‘sitting’, which has been considered, and others may want to share their experiences over 3 ‘sittings’.

***Withdrawal***

Parents or carers, and the autistic participants or participants with ID will be able to end any part of the visit or end the entire visit early if necessary. Participants will be allowed to withdraw before, during and up to two weeks following their narrative interview. During the initial screening assessment (SDQ/DBC), parents and carers and autistic participants or participants with ID will be reminded of each stage of the narrative interviews, using the topic guide and communication booklet, prior to starting and will be given the opportunity to change their minds about participating in any or all portions of the narrative interviews. The parents and carers and autistic participants or participants with ID will be reinformed prior to the start of each new stage of the narrative interview to ensure that they had the information needed to decide to participate in only some or all aspects of the study. Researchers as well as parents, carers, and participants will be allowed to stop or modify any task while in progress (e.g., the participant became too distressed). If participants become too distressed, they will be signposted to relevant support to ensure that harm is minimised.

***Incentives***

A £30 shopping voucher will be offered for 1–2 ‘sittings’ of the narrative interview per participant and a £100 shopping voucher will be offered for 3 ‘sittings’ of narrative interviews per participant. For all CYP who take part there will be a certificate of participation, social story book on Taking Part in Research designed and written by Mrs Williams and a sensory goodie bag in place of the shopping voucher, the costs will not exceed the amount of the shopping voucher. Travel for participation in the study will be reimbursed. 

***Data analysis***

CYP will share their story through narrative play, whereas parents/carers will tell their story verbally, and this will be captured through video, photographs, and voice recordings. Parents may be present during their CYP's interviews primarily in a supportive capacity to help facilitate communication and reduce anxiety, particularly for younger children or those with more significant communication needs. In the analysis, the primary data will be the CYP's direct communications (verbal, play-based, creative expressions); parent contributions during the CYP's interview will be coded separately and analysed as contextual/supporting data, we will clearly distinguish between direct child data and parental interpretations or additions and the separate parent interviews will provide their own distinct dataset. This approach allows us to prioritise the CYP's voice while acknowledging the supportive role parents may play in facilitating communication, particularly for CYP with more complex needs.

All data will be transcribed following the recording of each narrative interview. The initial review of the transcripts may highlight new or emerging themes that were not initially considered, and this will be reflected in future narrative interviews. Transcribing the data will be a lengthy process and time has been allocated for this. Narrative analysis will be used to analyse findings using topical stories. This will allow the research team to review the data beyond just the words said but how participants expressed themselves as well as their thoughts, feelings and experiences. Additionally, the narrative analysis will consider how the participants formed their narratives. The narrative analysis will focus on the overall story told by participants and organise the constructs of the narrative. Once narrative analysis is coded, the research team will review the participants’ story and see how individual narratives compare and diverge. We will first analyse the data collectively to identify common themes across both groups, then conduct a comparative analysis to identify any distinct needs or experiences specific to either autism or intellectual disability. This approach will allow us to develop both universal recommendations for improving mental health services for neurodevelopmental conditions broadly, as well as condition-specific recommendations where warranted. Narrative analysis for condition specific narrative interviews will be conducted in 6 steps: 1) code narrative blocks, 2) group and read by live-event, 3) create a nested story structure, 4) delve into the story structure, 5) compare across story structure and 6) tell the narrative. Preliminary results will be shared and discussed prior to the study’s full completion, where appropriate.

## Ethics and consent

### Ethics

The study received full ethical approval from the University of Warwick Biomedical and Scientific Research Ethics Committee (BSREC) [75/23-24], on Thursday, 21 ^st^ March 2024.

### Informed consent

The research team will share a social story booklet and animation video ( Williams, 2024) with autistic CYP and CYP with an ID. The video will include examples of other researchers facilitating narrative interviews; informed consent will be recorded in writing on pictorial consent forms. Informed written consent will be obtained from parent/carers for all minors under the age of 18 following their reading of the participant information sheet. Assent will be sought from all minors. If parents or carers are unsure if the person, they care for has capacity to consent, they will be advised to contact the research team. The research team can provide advice and give parents an assessment of capacity protocol to help with their decision-making. For participants who minors, the parent or caregiver will act as a personal or nominated consultee. This involves advising the research team on the best interests of the participant. One month before the study commences, we will ask parents and carers to discuss the research study with the person they care for in the most suitable way for their needs so that the participant has information about the study. Parents or carers can also request accessible picture or symbol, animated guides to help explain the research study to the person they care for. These guides can be tailored to each participant, for example, by using either the Picture Exchange Communication System symbols or Makaton images. We can also tailor these guides by adding personalised images. This could include the researcher attending the visit, family members who will be present, or a photo of the family home. Parent/carers will complete written consent for their participation in the narrative interviews.

## Dissemination

Findings will be presented at relevant conferences, in person or remotely through video conference, alongside an accessible written report and animated video. A full report of the study will be completed and will be accessible through the University of Warwick.

Academic: Association for Child and Adolescent Mental Health Conference, New Zealand; European Association for Mental Health in Intellectual Disability; Protocol- Wellcome Open Research and Study Findings- in the Association for Child and Adolescent Youth Mental Health journal.

Non-academic: Mental Health Support Team Webinars; Youth Advisory Groups/Boards across England; NHS England collaboration with DFE annual Events; The Cerebra Network and relevant ARC West Midlands collaboration events, local charities such as Autism East Midlands and community groups including Sensory Learning & Play C.I.C; and to local parliamentary members.

## Data Availability

No data are associated with this article. Open Science Framework: A Protocol to examine the needs and experiences of autistic children/young people or children/young people with an intellectual disability when receiving support for mental health difficulties from child and adolescent mental health services. https://doi.org/10.17605/OSF.IO/YT2XS ( Williams & Tuomainen, 2025). This project contains the following extended data: Childrens Consent Form V2 2024-03-09 19_55_59 Consent form PARENTS_CARERS V2 Episodic Narrative interview topic guide Participant Information Sheet_Parent_Carer v2 Taking Part (Teen) PIS Taking Part Book (Child) PIS Data are available under the terms of the Creative Commons Zero "No rights reserved" data waiver (CC0 1.0 Public domain dedication). Not applicable as the paper is a study protocol.
