# Supplementary material for: A Protocol to examine the needs and experiences of autistic children/young people or children/young people with an intellectual disability when receiving support for mental health difficulties from child and adolescent mental health services
Source: Wellcome Open Res. 2025 Jun 25;10:143. Originally published 2025 Mar 19. [Version 2] doi: 10.12688/wellcomeopenres.23597.2 (PMC12501585; doi:10.12688/wellcomeopenres.23597.2)
Supplement: Peer review history

# Reviewer report: Reviewer response for version 2

**DOI:** 10.21956/wellcomeopenres.27055.r133379

**Published:** 2025-10-06

**Authors:** Musa Sami

**Affiliations:** 1 National Health Service, England, UK

This is an important study looking at the experience of children and young people with autism and intellectual disability through UK services.

 The experience of the child is foregrounded and the experience of parents is also collected. Multiple techniques are used to engage children in order to conduct the narrative synthesis. The aims and objectives are clear and relevant. Although a small sample size (7-12) this is appropriate for this study design where depth is required. I wish to congratulate the authors on the design of this study.

 This is an extremely important area of research and it is clearly a difficult area to conduct research in. In particular engagement in service design and delivery from the perspective of the child is a fundamental question which is often overlooked. In such settings (from clinical and research experience) as the authors allude to the experience of children and young people is often overlooked for the view of the professionals and parents/caregivers. Hence services are designed for parents and professionals rather than putting the child at the heart of care. This is also noted in the research literature (caregiver, professional, researcher perspectives on services are well represented, there is very sparse work on the perspective of the child). Hence it is important to undertake this study. 

 I am happy to approve the paper, however I would suggest the following comments:

 1. I am unable to find any discussion in the introduction as to which studies have examined this question to date. There is actually sparse literature on specifically the child's perspective (most literature tends to focus on parent's, professionals or child givers perspectives or perspectives of adult patients) nonetheless a brief review on what has been done and how this study builds on this, differs from this would be useful.

 See for example: Malone et al 2025: doi: 10.1007/s10803-025-07044-y, O hagan et al (2024) doi: https://pubmed.ncbi.nlm.nih.gov/37871276/. I am unaware of whether any such work has been done in the UK but clearly this is a substantial issue here in Britain. 

 2. There should be a test for when data saturation is reached - to determine the exact endpoint when this has been met

 I am looking forward to seeing the results of this research

Is the study design appropriate for the research question?

Yes

Is the rationale for, and objectives of, the study clearly described?

Yes

Are sufficient details of the methods provided to allow replication by others?

Yes

Are the datasets clearly presented in a useable and accessible format?

Yes

Reviewer Expertise:

Psychiatry, clinical practice and service design

I confirm that I have read this submission and believe that I have an appropriate level of expertise to confirm that it is of an acceptable scientific standard.

## References

[1] : Saturation in qualitative research: exploring its conceptualization and operationalization. *Quality & Quantity* .2018;52(4) : 10.1007/s11135-017-0574-8 1893-1907 10.1007/s11135-017-0574-8 PMC599383629937585

# Reviewer report: Reviewer response for version 2

**DOI:** 10.21956/wellcomeopenres.27055.r127729

**Published:** 2025-09-05

**Authors:** David S Mandell

**Affiliations:** 1 University of Pennsylvania Perelman School of Medicine, Philadelphia, Pennsylvania, USA

The proposed study would conduct semi-structured interviews with between 7 and 12 autistic people with intellectual disability, and 7 to 12 caregivers of these children. The goal is to understand the challenges these individuals have in accessing mental health services.  The investigator proposes a narrative analysis of the resulting data. 

 The question under study is a critical one, as this population often is denied care because traditional children's mental health service providers don't have training in working with this group and tend to turf referred individuals to developmental disabilities services, which often don't have adequate mental health training. 

 While the area of study is important, there are many holes in the proposal that make it difficult to understand what data will be collected and how the study will advance knowledge in this area. Briefly:

 1. The introduction and rationale do not cite the growing body of literature in this area that have applied both quantitative and qualitative methods to understanding the difficulties this group has in accessing care. See for example, King et al, 2020; Pemovska et al., 2024; Drahota et al., 2023 (among many other studies). 

 2. While obtaining children and caregivers' perspectives is critically important, the voice of community providers is missing. They often are underpaid, undertrained, and have lots of anxiety about treating autistic youth. 

 3. Objective 1 is a quantitative question and seems like it would be better answered by survey or record review.

 4. the rational for so many interviews per subject is unclear. It seems that children could be interviewed for as much as 4.5 hours over three sittings, which seems excessive. I would argue that expanding the sample size would be more useful. 

 5.  I don't understand how the play activities are incorporated into answering the questions of interest. 

 6. The topics for the interview seem to be a mix of interview techniques (develop rapport) with phrases that don't provide insight into what will be asked (mental health difficulty). The questions about clinicians' confidence and knowledge seems like it would better answered by the clinicians. It could be that i am misunderstanding these topics and prompts, in which case the investigators should provide more detail, potentially turning these into sentences for ease of review. 

 7. the age span of 5-16 is quite wide given the topic. I don't understand how this interview works with a 5-year old with autism and intellectual disability. 

 8. I think it is premature to suggest that the findings will lead to universal recommendations to improve mental health services for this group. I would first want to see information from clinicians, replication of findings, and triangulation with quantitative data as well,

Is the study design appropriate for the research question?

Partly

Is the rationale for, and objectives of, the study clearly described?

Partly

Are sufficient details of the methods provided to allow replication by others?

No

Are the datasets clearly presented in a useable and accessible format?

Not applicable

Reviewer Expertise:

I am a autism researcher who focuses specifically on systems of care for these individuals.

I confirm that I have read this submission and believe that I have an appropriate level of expertise to state that I do not consider it to be of an acceptable scientific standard, for reasons outlined above.

# Reviewer report: Reviewer response for version 2

**DOI:** 10.21956/wellcomeopenres.27055.r126634

**Published:** 2025-07-23

**Authors:** Ayomipo Amiola

**Affiliations:** 1 Hertfordshire Partnership University NHS Foundation Trust, Hatfield, England, UK

I have now read the revised copy and the suggestions I made previously have been incorporated into the draft. I think the protocol (and indeed the research) will contribute to the evidence base and hopefully improve the outcomes of children and young people with ID and or ASD accessing child and adolescent mental health services.

Is the study design appropriate for the research question?

Yes

Is the rationale for, and objectives of, the study clearly described?

Partly

Are sufficient details of the methods provided to allow replication by others?

Partly

Are the datasets clearly presented in a useable and accessible format?

Not applicable

Reviewer Expertise:

ID, ASD, Psychiatry

I confirm that I have read this submission and believe that I have an appropriate level of expertise to confirm that it is of an acceptable scientific standard.

# Reviewer report: Reviewer response for version 1

**DOI:** 10.21956/wellcomeopenres.26033.r123537

**Published:** 2025-05-28

**Authors:** Ayomipo Amiola

**Affiliations:** 1 Hertfordshire Partnership University NHS Foundation Trust, Hatfield, England, UK

This is the protocol for a qualitative study of the needs and experiences of children and young people with ID and or autism who are accessing Child and adolescent mental health services due to mental health difficulties. This research will provide insight into the challenges this group faces and would also promote research involvement. Hopefully, by adding to the evidence base it can help shape the delivery of mental health services to children and young people with ASD and or ID.

 I have put below my comments to the authors.

 Thanks for this study investigating the needs and experiences of children and young people with autism and or ID who have coexisting mental health difficulties and are accessing mental health services.

**Introduction**

1. It would be helpful to clarify in the beginning if this study includes individuals with comorbid ASD and ID or children and young people with either one or both conditions and subsequently be consistent.
2. I think it will be good to also note in the introduction section about the major aetiological and diagnostic overlaps between both conditions including the risk they pose for developing acquired mental illnesses.
3. Since this is qualitative research, the point about generalisability is assumed and may be better left to the conclusion section following the completion of the study.

**Rationale**

1. You should cite the sentence about cyp with ASD or ID being refused access to generic mental health service and instead being signposted to specialist ID or ASD services. The sentence about diagnostic overshadowing is important in this connection, but Javaid et al’s work is a case report on a 54 year old man.
2. Also, while access to care is very important for this group, it does not appear straightforward how a qualitative study of the needs and experiences will improve access to care or necessarily improve outcomes, so you may want to flesh this out a bit.
3. It may be helpful to draw upon previous work in this area, then extend the discussion.
4. “It is *palpable”* that if clinicians intervened early, then the individual’s mental health may not have deteriorated. Did you mean to write “probable”?

**Research aims/questions/objectives**

1. The research objectives should not be a rephrasing of the research aim Instead the objectives should set out in specific, measurable and detailed points how the aim will be accomplished.

E.g. Aim: To understand the experience of children and young people with ASD and ID who access mental health services.

 Objectives: 1. To understand the length of time between referral and first assessment. 2. To understand the barriers patients face in accessing mental health services. 3. To understand the level of satisfaction patients derive from services, et cetera.

**Methods**

1. Since you have quoted “sitting”, it may help to explain what this means and how you are using it differently from its normal everyday language especially since one will assume that reasonable adjustments will have to be made to account for the cognitive differences in this group such as breaks, time outs, et cetera.
2. You may also want to specify in this protocol, the alternative methods of communication that you intend to use.
3. I have noted above the need for clarity in terms of the study participants. The eligibility criteria states, “Participants will include autistic CYP with ID aged 5–16” however the inclusion criteria states, “a clinical diagnosis of autism and/or ID”.
4. In terms of the procedure, there are three possible locations: the individual’s home, online or a neutral setting chosen by the participant. Perhaps a sentence should be provided to clarify the impact of varying environments on the data obtained? Prior to when the study is completed, results are available and discussed.  

Is the study design appropriate for the research question?

Yes

Is the rationale for, and objectives of, the study clearly described?

Partly

Are sufficient details of the methods provided to allow replication by others?

Partly

Are the datasets clearly presented in a useable and accessible format?

Not applicable

Reviewer Expertise:

ID, ASD, Psychiatry

I confirm that I have read this submission and believe that I have an appropriate level of expertise to confirm that it is of an acceptable scientific standard, however I have significant reservations, as outlined above.

# Response

**Published:** 2025-06-12

**Authors:** Naomi Williams

**Affiliations:** Health Sciences, University of Warwick Medical School Division of Metabolic and Vascular Health, Warwick, England, UK

Dear Reviewer, Thank you for taking the time to review this protocol. Please see responses below. 

**Introduction**

**1. It would be helpful to clarify in the beginning if this study includes individuals with comorbid ASD and ID or children and young people with either one or both conditions and subsequently be consistent.**

 Thank you for this helpful suggestion. I have now clarified in the introduction that the study includes autistic children and young people (CYP) with intellectual disability (ID), either as co-occurring conditions or as standalone diagnoses, and this has been made consistent throughout the paper.

**2. I think it will be good to also note in the introduction section about the major aetiological and diagnostic overlaps between both conditions including the risk they pose for developing acquired mental illnesses.**

 This point has been incorporated into the rationale section of the introduction as part of the response to Reviewer 1. The overlapping characteristics of autism and ID, and the associated increased vulnerability to developing mental health difficulties, are now discussed more explicitly.

**3. Since this is qualitative research, the point about generalisability is assumed and may be better left to the conclusion section following the completion of the study.**

 Thank you. There is currently no reference to generalisability in the introduction. This point will be addressed in the conclusion section once the study has been completed.

**Rationale**

**1. You should cite the sentence about CYP with ASD or ID being refused access to generic mental health service and instead being signposted to specialist ID or ASD services. The sentence about diagnostic overshadowing is important in this connection, but Javaid et al’s work is a case report on a 54 year old man.**

 Thank you for highlighting this. I have now included an appropriate citation to support this point and have replaced the case report reference with a more relevant source.

**2. Also, while access to care is very important for this group, it does not appear straightforward how a qualitative study of the needs and experiences will improve access to care or necessarily improve outcomes, so you may want to flesh this out a bit.**

 I appreciate this feedback. I have now expanded the rationale to better articulate the value of a qualitative approach. A study capturing the lived experiences and perceived needs of this population and their families can reveal specific barriers, such as communication difficulties, service inflexibility, or sensory environments, that are often overlooked in quantitative research. Identifying these nuanced factors can inform the development of more accessible, responsive, and equitable services, supporting earlier intervention, reducing disengagement, and ultimately improving outcomes. Additionally, this approach aligns with national priorities around co-production and person-centred care in service design.

**3. It may be helpful to draw upon previous work in this area, then extend the discussion.**

 Thank you for this helpful suggestion. I have now referenced the studies by Whittle et al. (2018), which highlight the system-level barriers and communication challenges faced by autistic children and those with intellectual disability, and this has been used to extend the rationale and discussion.

**4. “It is palpable” that if clinicians intervened early, then the individual’s mental health may not have deteriorated. Did you mean to write “probable”?**

 Yes, thank you for catching this. The word “palpable” has now been replaced with “probable.”

**Research Aims/Objectives**

**1. The research objectives should not be a rephrasing of the research aim. Instead, the objectives should set out in specific, measurable and detailed points how the aim will be accomplished.**

 Thank you for this important clarification. I have revised the research objectives accordingly to reflect distinct, measurable aims:

1. To examine the time intervals between key stages in the mental health care pathway, onset of difficulty, referral, triage, assessment, intervention, and discharge for autistic CYP and/or CYP with ID.
2. To identify and explore barriers faced by autistic CYP and/or those with ID in accessing timely and appropriate mental health services.
3. To evaluate the perceived and actual impact of mental health services on outcomes for autistic CYP and/or those with ID.

**Methods**

**1. Since you have quoted “sitting”, it may help to explain what this means and how you are using it differently from its normal everyday language especially since one will assume that reasonable adjustments will have to be made to account for the cognitive differences in this group such as breaks, time outs, et cetera.**

 Thank you. I have clarified that “sitting” refers to one interview session and that this will be flexibly structured to allow for appropriate reasonable adjustments such as breaks, pauses, or rescheduling if needed.

**2. You may also want to specify in this protocol the alternative methods of communication that you intend to use.**

 This has now been added. Alternative communication methods will include Makaton, symbols, pictures, photographs, and social stories to ensure accessibility and inclusion.

**3. I have noted above the need for clarity in terms of the study participants. The eligibility criteria states, “Participants will include autistic CYP with ID aged 5–16” however the inclusion criteria states, “a clinical diagnosis of autism and/or ID”.**

 Thank you for pointing this out. I have now corrected the wording to read: “Participants will include autistic CYP and/or those with ID aged 5–16.”

**4. In terms of the procedure, there are three possible locations: the individual’s home, online or a neutral setting chosen by the participant. Perhaps a sentence should be provided to clarify the impact of varying environments on the data obtained? Prior to when the study is completed, results are available and discussed.**

 Thank you for this helpful suggestion. I have now added the following sentence to address this point:

 “Consideration has been given to how each environment may influence participant responses. For instance, conducting interviews in the home may enhance comfort and openness while also reducing the stress associated with travel and other commitments. A neutral venue may help minimise distractions and familial influence but could increase anxiety or sensory overwhelm for some participants. Online participation, while potentially limiting the depth of responses due to the virtual format, may offer greater convenience, particularly for parents, by allowing engagement at more flexible times, such as when children are asleep or occupied.”

# Reviewer report: Reviewer response for version 1

**DOI:** 10.21956/wellcomeopenres.26033.r121005

**Published:** 2025-04-08

**Authors:** Vaso Totsika

**Affiliations:** 1 University College London, London, England, UK

In this protocol for a planned qualitative study on the experience of mental health services of autistic children or children with intellectual disability, I would like to draw the authors' attention to

 1. **The issue of setting/exposure and how this ties in with the recruitment criteria: I**n England there is wide variation in the names and set up of services that provide support to these children. It is unclear currently whether the authors intend to focus just on CAMHS or any mental health support services, and also whether they are looking for children/families currently referred to specialist CAMHS or any CAMHS. Differences are substantial and the set up of services differs by geographical area, as well. Therefore, findings may just reflect specific areas or the experience of specific services. I wondered  if sampling/findings would make more sense if authors restricted either the definition of the setting or the geographical area relevant to the investigation. Finally, the requirement for participants to have both experience of an unsuccessful referral to CAMHS and an active referral might be too restrictive when it comes to sampling. 

 2. **Ethics:** Given participants are recruited who are active 'patients' of NHS services and because of their experience of being a patient in NHS services, I wondered if IRAS was a more suitable place to go for ethical review? I appreciate the researchers will not be recruiting from the NHS. Also, please revise the section on consents/assents to be more in line with the legal framework for doing research with children (MCA).

 3. It would be helpful if the rationale for the study explained why it focuses on autistic children and children with intellectual disability (and not on one of these groups only, or more neurodevelopmental conditions), whether results will be analysed together or separately (by condition) and how the analysis decision will be linked to clinical implications (see also comment 1 about variation in service types, where some services are specific to children with LD, some for all NDDS etc). 

 4. **The role of the parent in the child interviews: **If parents are present during their child's interviews, how will the researchers treat the data from the parent in the analysis?

Is the study design appropriate for the research question?

Yes

Is the rationale for, and objectives of, the study clearly described?

Partly

Are sufficient details of the methods provided to allow replication by others?

Partly

Are the datasets clearly presented in a useable and accessible format?

Not applicable

Reviewer Expertise:

intellectual disability and autism

I confirm that I have read this submission and believe that I have an appropriate level of expertise to confirm that it is of an acceptable scientific standard, however I have significant reservations, as outlined above.

# Response

**Published:** 2025-04-24

**Authors:** Naomi Williams

**Affiliations:** Health Sciences, University of Warwick Medical School Division of Metabolic and Vascular Health, Warwick, England, UK

Thank you for your thoughtful review of our protocol. We appreciate your constructive feedback and have addressed each point below.

**1. Setting/Exposure and Recruitment Criteria** We acknowledge the critical point about service variation across England. To clarify: Our study focuses specifically on Child and Adolescent Mental Health Services (CAMHS) rather than all mental health support services. We recognise the geographical variations in CAMHS setup and delivery, which is why our recruitment strategy deliberately seeks participants from diverse geographical areas across England to capture this variability. Regarding recruitment criteria, we appreciate your concern about requiring both unsuccessful and active referrals. If this was unclear in our protocol, to clarify, participants need to have **either** current/previous experience receiving psychological therapy through CAMHS **or** past experience of unsuccessfully accessing CAMHS. This "or" criterion provides flexibility to capture diverse experiences, including both successful and unsuccessful pathways, without requiring participants to have experienced both scenarios. **2. Ethics** Thank you for raising the point about IRAS (Integrated Research Application System) review. We carefully considered the appropriate ethical approval pathway for this study. Since our recruitment does not occur through NHS services (participants are recruited via social media, conferences, and community organisations rather than through NHS patient lists), and we are not accessing NHS patient data or conducting the research on NHS premises, BSREC approval was determined to be appropriate.

- We appreciate your concern about MCA compliance. We believe our protocol does align with the legal framework, as it includes
- Obtaining informed written consent from parents/carers for all minors under 18
- Seeking assent from minors who can understand information about the study
- A process for assessing capacity when parents/carers are unsure
- Provisions for parents/carers to act as personal or nominated consultees for participants who cannot consent
- Adaptations to make information accessible (including picture systems, Makaton images and symbols.)

**3. Study Population Rationale** We appreciate the suggestion to clarify our rationale for including both autistic children and children with intellectual disability. Our decision was based on:

1. The significant clinical overlap and co-occurrence of these conditions (approximately one-third, 37.9% of autistic children also have intellectual disability).
2. The shared challenges both groups face in accessing appropriate mental health support.
3. The similar adaptations often needed to make psychological therapies accessible to both populations.

In our analysis, we will first analyse the data collectively to identify common themes across both groups, then conduct a comparative analysis to identify any distinct needs or experiences specific to either autism or intellectual disability. This approach will allow us to develop both universal recommendations for improving mental health services for neurodevelopmental conditions broadly, as well as condition-specific recommendations where warranted. **4. Role of Parent in Child Interviews** Thank you for this important methodological question. Parents may be present during their child's interviews primarily in a supportive capacity to help facilitate communication and reduce anxiety, particularly for younger children or those with more significant communication needs. In our analysis:

- The primary data will be the child's direct communications (verbal, play-based, creative expressions)
- Parent contributions during the child's interview will be coded separately and analysed as contextual/supporting data
- We will clearly distinguish between direct child data and parental interpretations or additions
- The separate parent interviews will provide their own distinct dataset

This approach allows us to prioritise the child's voice while acknowledging the supportive role parents may play in facilitating communication, particularly for children with more complex needs. We appreciate the thoughtful review and believe these clarifications strengthen our protocol, as such the protocol has been updated with any additions highlighted in green and any specific areas raised for your information highlighted in yellow. We would be happy to provide further information if needed.
